# Polycomb Repressive Complex 2 (PRC2) Restricts Hematopoietic Stem Cell Activity

**DOI:** 10.1371/journal.pbio.0060093

**Published:** 2008-04-15

**Authors:** Ian J Majewski, Marnie E Blewitt, Carolyn A de Graaf, Edward J McManus, Melanie Bahlo, Adrienne A Hilton, Craig D Hyland, Gordon K Smyth, Jason E Corbin, Donald Metcalf, Warren S Alexander, Douglas J Hilton

**Affiliations:** 1 The Walter and Eliza Hall Institute of Medical Research, Parkville, Victoria, Australia; 2 The Department of Medical Biology, The University of Melbourne, Parkville, Victoria, Australia; Baylor College of Medicine, United States of America

## Abstract

Polycomb group proteins are transcriptional repressors that play a central role in the establishment and maintenance of gene expression patterns during development. Using mice with an N-ethyl-N-nitrosourea (ENU)-induced mutation in *Suppressor of Zeste 12* (*Suz12*), a core component of Polycomb Repressive Complex 2 (PRC2), we show here that loss of Suz12 function enhances hematopoietic stem cell (HSC) activity. In addition to these effects on a wild-type genetic background, mutations in *Suz12* are sufficient to ameliorate the stem cell defect and thrombocytopenia present in mice that lack the thrombopoietin receptor (*c-Mpl*). To investigate the molecular targets of the PRC2 complex in the HSC compartment, we examined changes in global patterns of gene expression in cells deficient in Suz12. We identified a distinct set of genes that are regulated by Suz12 in hematopoietic cells, including eight genes that appear to be highly responsive to PRC2 function within this compartment. These data suggest that PRC2 is required to maintain a specific gene expression pattern in hematopoiesis that is indispensable to normal stem cell function.

## Author Summary

The chromatin environment that surrounds a gene heavily influences the gene's transcriptional activity. Specific modifications on histone tails serve as signposts for the basal transcriptional machinery, reflecting a cell's developmental history and identifying genes that should be actively transcribed and those that must be repressed. Polycomb group proteins are involved in large, multiprotein complexes that catalyse the post-translational modification of histones. The disruption of these complexes induces wholesale changes in gene expression, a scenario commonly seen in diseases such as cancer. We have investigated the role of Polycomb group proteins during blood cell formation: in stem cells, progenitor cells, and mature blood cells. Using a variety of functional assays, we demonstrate an important role for Polycomb group proteins in restricting the activity of hematopoietic stem cells. To define the molecular targets of the complex, we examined gene expression profiles in cells with impaired expression of Polycomb group proteins. This analysis identified a set of target genes within the hematopoietic compartment that was distinct from those defined in embryonic stem cells and fibroblasts. This study provides new insights into the role of these proteins during hematopoiesis, and suggests a novel mechanism by which they might contribute to leukaemia.

## Abstract 3

Epigenetic modifications are central to the maintenance of cellular identity and are dynamically regulated during differentiation. We addressed the role of Polycomb group proteins during hematopoiesis and define a series of genes that are highly responsive to Polycomb dysfunction.

## Introduction

*Suppressor of Zeste 12* (*Suz12*) was identified in a genetic screen performed in *Drosophila* to define transcriptional repressors [1]. Biochemical studies subsequently identified Suz12 as a component of a multimeric protein complex, termed Polycomb Repressive Complex 2 (PRC2), which is responsible for di- and tri-methylation of histone 3 at lysine 27 (H3K27) [2–5]. The core components of PRC2 are conserved between fly and vertebrates; in addition to Suz12, they include the methyl-transferase Enhancer of Zeste 2 (Ezh2) and various forms of the Embryonic Ectoderm Development protein (Eed) (reviewed in [6]). Genome-wide location analysis—performed in mouse, human, and fly—confirmed that PRC2 components and tri-methylated H3K27 (H3K27-3Me) are enriched within the promoters of transcriptionally repressed genes [7–14]. Disruption of the complex has profound consequences during development and in human disease, which illustrates the important role epigenetic marks play in maintaining appropriate patterns of gene expression.

Many insights into the mechanism of PRC2 action have come from studies focused on its role in Homeobox gene repression in *Drosophila*. Work with the *Ultrabithorax* promoter demonstrated that repression mediated by PRC2 involves the recruitment of a distinct Polycomb complex, termed Polycomb Repressive Complex 1 (PRC1) [2,5]. Later studies confirmed that the *Drosophila* protein Polycomb (Pc), a core component of PRC1, binds with high affinity to H3K27-3Me via its chromodomain [15,16]. Because H3K27 methylation is performed by PRC2, these findings suggested a hierarchical relationship between the two complexes, in which PRC2 initiates silencing by targeting PRC1 to specific regions of chromatin (reviewed in [6]). The relationship between PRC2 and PRC1 is further complicated in vertebrates where many Polycomb group homologs are present [17]. Indeed, the hierarchy amongst distinct Polycomb group complexes and their dynamic composition and interaction in physiological processes, such as X-inactivation, remain incompletely understood [18]. Newly identified links between PRC2 and other epigenetic regulators, such as DNA methyl-transferase and noncoding RNA molecules (ncRNA), suggest that PRC2 coordinates a variety of processes that function in concert to initiate and maintain the repressive chromatin state [19,20].

Given that PRC2 regulates numerous target genes, it is perhaps not surprising that disruption of the complex has major implications during the early stages of development, where the correct temporal and spatial control of gene expression is critical. Murine models of PRC2 deficiency have demonstrated that each component is absolutely required for embryonic development [21–23]. The analysis of embryos that lack PRC2 components has revealed deficiencies at implantation and early post-implantation stages in development, which is consistent with the involvement of PRC2 in pathways that influence cellular proliferation [24–26]. Further investigation of PRC2 function in the adult mouse has been restricted to the use of conditional alleles, which have been generated for *Ezh2* [27], and viable hypomorphic alleles that include *Eed^1989^* [21].

The study of Polycomb group proteins in vertebrate hematopoiesis has largely focused on PRC1 components, the best-characterised amongst these being Bmi-1. Bmi-1 is a critical regulator of self-renewal in hematopoietic stem cells (HSCs) [28–30] that mediates its effect in part through control of the *Ink4a-Arf* locus [29,31,32]. In comparison, relatively little is known about the activity of PRC2 within the HSC compartment, although several observations suggest that perturbation of PRC2 influences HSC biology. Hypomorphic alleles of *Eed* have demonstrated a critical role for PRC2 in restricting the proliferation of early lymphoid and myeloid progenitors [33,34]. In this context, the function of PRC2 appears to oppose the activity of PRC1; however, the precise molecular targets that contribute to this phenotype remain unknown [33]. Proliferative defects have not been reported in mice that lack Ezh2 within the hematopoietic compartment, although this finding may be complicated by impaired B cell and T cell maturation [27,35]. Further evidence of a role for PRC2 in the stem cell compartment has come from the finding that forced expression of Ezh2 appears to prevent the exhaustion of HSCs during serial transplantation [36].

Thrombopoietin (Tpo) is the primary regulator of platelet production in vivo [37,38]. Deletion of the *Tpo* gene, or the Tpo receptor (*c-Mpl*), in mice results in a severe reduction in platelet count [39–41], and mutations that disrupt the Tpo/c-Mpl pathway are the major cause of the rare human disorder congenital amegakaryocytic thrombocytopenia [42,43]. Signalling through c-Mpl also supports the development of long-term HSCs [44], and impaired signalling through the receptor is associated with functional deficiencies in HSCs and progenitor cells in both mice and humans [41,45–47]. We have performed a large-scale ENU mutagenesis screen to identify mutations that rescue platelet production and HSC function in *c-Mpl^–/–^* mice. This approach has previously identified mutations in *c-Myb* that result in supra-physiological platelet production [48]. Herein we describe the isolation of a mutation in *Suz12*, identified in the platelet 8 (PLT8) pedigree, which causes a global reduction in the abundance of PRC2. Impairment of PRC2 in *Suz12^Plt8/+^* mice caused changes in steady-state hematopoiesis that were associated with enhanced HSC and progenitor cell activity.

## Results

### Genetic Mapping and Identification of the *Plt8* Mutation

An ENU mutagenesis screen was performed with *c-Mpl^−/−^* mice to identify mutations that suppress thrombocytopenia and/or stem cell defects. The average platelet count in *c-Mpl^–/–^* mice is 112 ± 78 × 10^6^/ml (mean ± standard deviation; *n* = 179). The founder of the PLT8 pedigree was identified among a population of G_1_ animals segregating ENU-induced mutations due to its unusually high platelet count (361 × 10^6^/ml), more than three standard deviations above the mean. The phenotype was found to be heritable and was therefore likely to be the result of a germline ENU-induced mutation.

**Figure 1:**
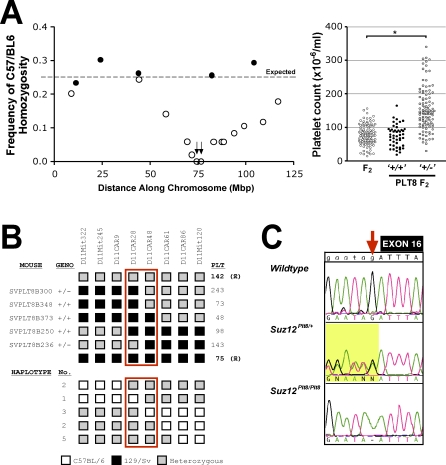
Genetic Mapping and Identification of the *Plt8* Mutation (A) 90 PLT8 F_2_ mice were genotyped with polymorphic microsatellite markers spread throughout the genome. The frequency of C57BL/6 homozygosity is plotted along the length of Chromosome 10 (filled circles) and Chromosome 11 (open circles). No mice were homozygous C57BL/6 across an interval on Chromosome 11 (arrows). PLT8 F_2_ mice that carried C57BL/6 DNA (‘+/–') at this position had a higher mean platelet count than wildtype mice (a control F_2_ population, *p < 0.002) or littermates that were homozygous 129/Sv (‘+/+'). (B) A 1.39-Mb candidate interval was defined between *D11CAR28* and *D11CAR48* (boxed in red). Genotyping is shown for individual recombinants (top panel); C57BL/6 homozygosity is represented by open boxes, heterozygous markers are gray and 129/Sv homozygosity is shown in black. A haplotype map is shown (bottom panel) that defines the same interval using the lethality phenotype; the number of mice with each haplotype is shown at left. (C) DNA was extracted from PLT8 mice with elevated platelet counts for sequence analysis. A single base pair deletion was identified in heterozygous mice (*Suz12^Plt8/+^*) and in homozygous tissue obtained from embryos (*Suz12^Plt8/Plt8^*) (red arrow). The deletion disrupts the splice acceptor site upstream of exon 16.

The *Plt8* mutation was generated on a C57BL/6 background and, for the purposes of mapping, was crossed to 129/Sv to generate C57BL/6:129/Sv F_1_ animals. F_1_ animals with an elevated platelet count (the PLT8 phenotype) were intercrossed to produce an F_2_ population for positional cloning. Initial results from the genome-wide scan localised the mutation to a 6.5-Mb interval on Chromosome 11, between *D11MIT245* and *D11MIT120*. There was a reduced frequency of C57BL/6 homozygosity at this position—below the expected frequency of 25%—which suggested the presence of a mutation that was lethal when homozygous (Figure 1A, at left). Analysis of the peripheral blood demonstrated that mice that carried C57BL/6 DNA at this position (as heterozygotes) had a higher mean platelet count when compared to mice that were 129/Sv homozygotes, or to a control F_2_ population, which suggested that this region harboured the *Plt8* mutation (Figure 1A, at right). Additional microsatellite markers were used to refine the *Plt8* candidate interval. An additional 531 PLT8 F_2_ mice were genotyped, and the candidate interval was reduced to 1.4 Mb between *D11CAR28* and *D11CAR48* (from base pair 79314902 to 80704402) (Figure 1B).

We sequenced the exons and splice sites of six genes within the candidate interval: *Suppressor of Zeste 12* (*Suz12*), *Cytokine receptor-like factor 3*, *Ring finger protein 135*, *Rhomboid veinlet-like protein 4*, *Zinc finger protein 207*, and *Cyclin-dependent kinase 5 activator 1 precursor*. A single base pair deletion was identified in a splice acceptor site of the 16th exon of *Suz12* (Figure 1C). The *Plt8* mutation was present in all affected PLT8 animals and was not identified in wild-type littermates, or other available mouse strains that included 129/Sv, C3H, and Balb/c.

### *Suz12^Plt8^* Fails to Complement a Null Allele of *Suz12*

A complementation test was performed to verify that the *Plt8* mutation impairs the functional activity of Suz12. Mice heterozygous for the *Plt8* mutation (*Suz12^Plt8/+^*) were mated to mice that carried a loss-of-function genetrap allele (*Suz12^502gt/+^*) to generate compound heterozygotes. The genetrap allele, herein referred to as *Suz12^502gt^*, has been characterised previously and shown to impair Suz12 function [23]. No compound heterozygotes (*Suz12^Plt8/502gt^*) were identified from 100 pups analysed (Table S1, chi squared *p*-value = 2.50 × 10^−7^), which indicated that *Suz12^Plt8/502gt^* mice die prior to weaning. Pasini and colleagues previously demonstrated that *Suz12^502gt/502gt^* embryos die approximately 8 d after fertilization [23]. Similarly, embryos homozygous for the *Plt8* mutation were reduced in number at embryonic day 8.5 (E8.5) and were smaller than wild-type and heterozygous embryos (unpublished data). These results strongly suggest that the *Plt8* mutation impairs *Suz12* function.

### The *Plt8* Mutation Impairs Suz12 mRNA Processing and Protein Production

**Figure 2:**
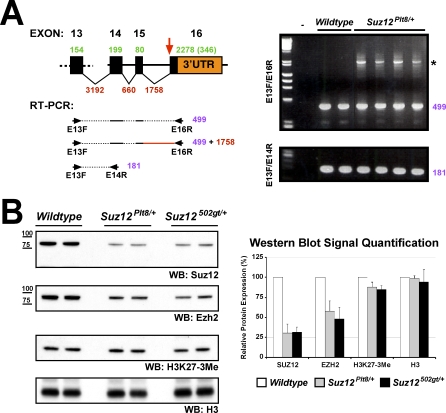
The *Plt8* Mutation Disrupts Production of the Suz12 mRNA and Protein (A) A schematic representation of part of the *Suz12* locus is shown to detail the intron and exon structure (at left). Primers were designed to flank the site of the mutation (red arrow), and RT-PCR was performed on cDNA prepared from bone marrow. An aberrantly spliced product (asterisk) was identified in cDNA prepared from *Suz12^Plt8/+^* mice that was not present in wild type. (B) Protein lysates were prepared from sex-matched embryonic day 12.5 (E12.5) embryos for analysis by Western blotting, which revealed that Suz12 and Ezh2 protein levels were reduced in *Suz12^Plt8/+^* embryos. Suz12 protein levels appeared equivalent in *Suz12^Plt8/+^* embryos and embryos heterozygous for the genetrap allele (*Suz12^502gt/+^*). Equivalent amounts of protein were run in each lane (20 μg), and histone H3 was used to verify equal loading. Western blot signal intensity was quantified using a densitometer; results represent the average of two independent experiments expressed as protein expression in *Suz12^Plt8/+^* (gray) and *Suz12^502gt/+^* (black) embryos relative to wild type (white, 100%).

*Suz12* mRNA and protein were analysed to determine the mechanism by which the *Plt8* mutation affects Suz12 function. Consistent with a deletion in the splice acceptor site of exon 16, an analysis of *Suz12* mRNA by reverse-transcriptase (RT)-PCR demonstrated aberrant splicing of the *Suz12* transcript in mice that carry the *Plt8* mutation (Figure 2A). A longer *Suz12* transcript was evident in cDNA from bone marrow and spleen of these mice, and DNA sequencing confirmed that the longer mRNA resulted from inappropriate inclusion of the 15th intron within the mature transcript. Inclusion of the 15th intron introduces a stop codon that is predicted to truncate 115 amino acids at the C terminus of the protein (Suz12ΔC115).

Suz12 protein levels were reduced in lysates prepared from *Suz12^Plt8/+^* embryos. There was no evidence of the predicted truncation product Suz12ΔC115 (Figure 2B), despite the fact that the anti-Suz12 antibody could detect the truncated protein when it was expressed exogenously in transfected fibroblasts (Figure S1). Ezh2 protein levels were also lower in *Suz12^Plt8/+^* embryos (Figure 2B), a finding consistent with previous reports that Suz12 influences the stability of other PRC2 components [23]. Impaired production of Suz12 and Ezh2 had only a modest effect on the total amount of H3K27-3Me in mutant embryos. Similar changes were evident in embryos that were heterozygous for the genetrap allele (Figure 2B), suggesting that the primary effects of both mutations are to reduce steady state levels of Suz12 protein.

### Genetic Impairment of Suz12 Enhances Platelet Production in *c-Mpl^–/–^* Mice

Analysis of peripheral blood confirmed that *Suz12^Plt8/+^ c-Mpl^–/–^* mice had a significantly increased platelet count (252 ± 54 × 10^6^/ml) when compared to *Suz12^+/+^ c-Mpl^–/–^* littermates (107 ± 54 × 10^6^/ml) (Table 1). Although mildly elevated, the increase in platelet count in *Suz12^Plt8/+^* mice on a *c-Mpl^+/+^* background was not statistically significant (Table 1). White blood cell numbers were also elevated in *Suz12^Plt8/+^* mice, independent of the *c-Mpl* genotype, due to an increased number of lymphocytes. Platelet volume, red cell count, and hematocrit were not affected by the *Plt8* mutation. Similar changes were evident in the peripheral blood of mice that carry the genetrap allele (*Suz12^502gt^*), which confirmed that the elevation in platelet count was a result of impaired Suz12 function (Table 1). The mixed genetic background of *Suz12^502gt/+^* mice is likely to account for variation in the magnitude of the changes. We also identified elevated platelet counts in mice that carried a null allele of *Ezh2* (93 ± 14 × 10^6^/ml in *Ezh2^+/+^ c-Mpl^–/–^* mice compared to 152 ± 14 × 10^6^/ml in *Ezh2^+/-^ c-Mpl^–/–^* littermates, *p* = 0.0095) [22], which further suggests that impairment of PRC2 underlies the phenotypic changes evident in *Suz12^Plt8/+^* mice.

**Table 1:**
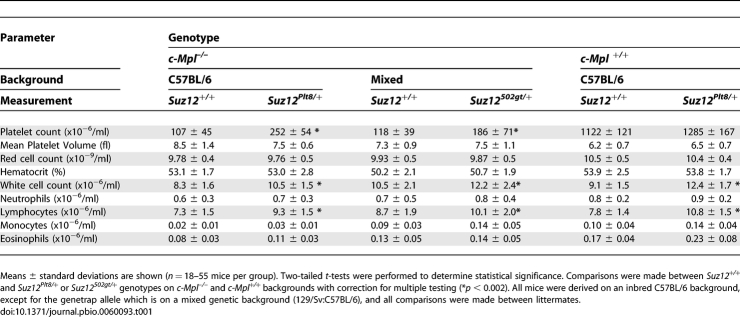
Peripheral Blood Profile of *Suz12^Plt8/+^* and *Suz12^502gt/+^* Mice

**Figure 3:**
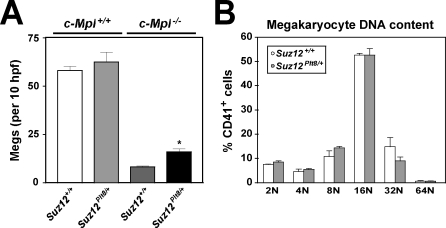
Megakaryocyte Number and DNA Content in PLT8 Mice (A) Megakaryocytes were counted on stained sections of bone marrow (600× magnification). The data represent the average number of megakaryocytes per 10 fields (hpf); 3–16 mice were scored for each genotype. Error bars represent the standard error of the mean and an asterisk has been used to denote statistical significance (*p<0.001). (B) The DNA content of wild-type and mutant megakaryocytes was analysed by flow cytometry using CD41 staining in combination with propidium iodide (*Suz12^Plt8/+^* mice *n* = 3, and controls *n* = 4). No statistically significant differences were observed.

Consistent with the elevation in platelet count, megakaryocyte numbers were increased in the bone marrow of *Suz12^Plt8/+^ c-Mpl^–/–^* mice, and no significant increase was seen in *Suz12^Plt8/+^ c-Mpl^+/+^* mice (Figure 3A). Histological examination demonstrated normal megakaryocyte morphology (unpublished data), and no differences in megakaryocyte DNA-ploidy were evident (Figure 3B).

### Hematopoietic Progenitor Cell Analysis in *Suz12^Plt8/+^* Mice

To characterize the hematopoietic progenitor cell compartment, in vitro colony assays were performed. Numbers of progenitor cells responsive to several stimuli appeared normal in *Suz12^Plt8/+^* mice (Table 2). Megakaryocyte progenitor numbers were slightly elevated in bone marrow and spleen cultures from *Suz12^Plt8/+^ c-Mpl^+/+^* mice; however, this difference was not statistically significant and was not evident in mice on a *c-Mpl^–/–^* background (Table 2).

**Table 2:**
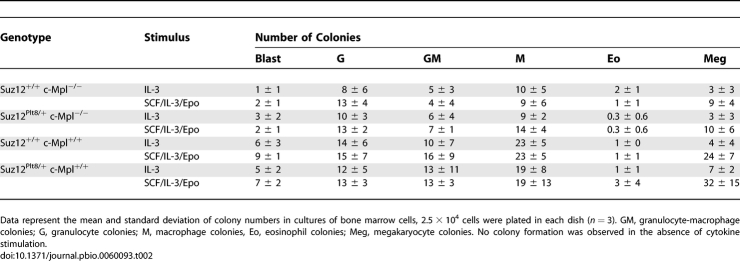
Hematopoietic Progenitors in *Suz12^Plt8/+^* Bone Marrow

**Figure 4:**
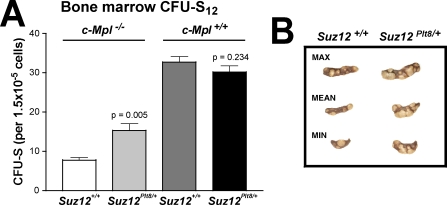
The *Plt8* Mutation Enhances CFU-S Frequency in *c-Mpl^–/–^* Mice (A) CFU-S frequency was assessed in bone marrow derived from *Suz12^Plt8/+^* mice and wild-type littermates. Irradiated recipients received 1.5 × 10^5^ nucleated bone marrow cells from *c-Mpl^–/–^* donors or 7.5 × 10^4^ cells from *c-Mpl^+/+^* donors. Data represent the mean of 4–6 mice of each genotype, and error bars show the standard error of the mean. Statistical significance was assessed using an unpaired *t*-test. (B) Representative spleens from recipients of *Suz12^Plt8/+^ c-Mpl^–/–^* and *Suz12^+/+^ c-Mpl^–/–^* bone marrow were photographed to detail the size and number of colonies (at right).

Multipotential hematopoietic progenitor cells can be quantified using their propensity to form colonies in the spleen of lethally irradiated mice; these colonies are referred to as colony-forming units spleen (CFU-S) [49]. In agreement with previous studies that detailed a reduction in stem cell function in *c-Mpl^–/–^* mice, these animals show a dramatic reduction in CFU-S compared with *c-Mpl^+/+^* mice (Figure 4) [45]. *c-Mpl^–/–^* mice that carry the *Plt8* mutation had a significantly increased number of CFU-S when compared to *Suz12^+/+^ c-Mpl^–/–^* littermates (Figure 4). This increase was not observed on a *c-Mpl^+/+^* background.

### Analysis of HSCs in *Suz12^Plt8/+^* Mice

**Figure 5:**
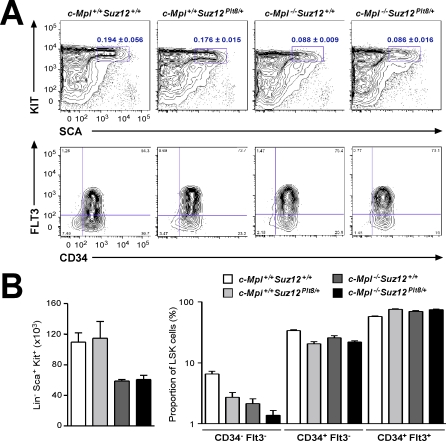
Immunophenotypic Analysis and Quantification of HSCs in *Suz12^Plt8/+^* Mice (A) The representation of LSK cells was assessed in whole bone marrow from *Suz12^Plt8/+^* mice and wild-type littermates. The percentage shown represents the mean (± standard deviation) of 4–7 mice of each genotype. Analysis of LT- and ST-HSCs was performed on lineage-depleted bone marrow. (B) The absolute number of LSK cells present in four leg bones (two femurs and two fibulas) was determined. The proportion of CD34^–^ Flt3^–^, CD34^+^ Flt3^–^ and CD34^+^ Flt3^+^ cells is shown for the LSK population, data represent the mean (± standard error) of 4–12 mice of each genotype.

The number of immunophenotypic HSCs was quantified to determine whether the stem cell compartment was expanded in *Suz12^Plt8/+^* mice. Consistent with previous reports of progenitor cell defects, the number of lineage marker negative (Lin^–^), Sca-1^+^, c-Kit^+^ (LSK) cells in the bone marrow was reduced in the absence of c-Mpl (∼2-fold, *p* < 0.001); however, there was no discernable difference in the total number of LSK cells between *Suz12^Plt8/+^* and *Suz12^+/+^* mice (Figure 5). The cell surface proteins CD34 and FMS-like tyrosine kinase 3 (Flt3) were used to subdivide the LSK population into long-term (LT) HSCs, short-term (ST) HSCs, and lymphoid primed multi-potent progenitors (LMPPs) [50]. *c-Mpl^–/–^* mice exhibit a marked reduction in the frequency of CD34^–^ Flt3^–^ LSK cells, the population that contains LT-HSCs, which is similar to results obtained with *Tpo* ^*–/–*^ animals [44]. Rather than being increased, the proportion of CD34^–^ Flt3^–^ LSK cells was slightly lower in *Suz12^Plt8/+^* mice, and this difference was more pronounced in *c-Mpl^+/+^* mice (*p* < 0.01) (Figure 5B). The increased frequency of lymphoid-biased progenitors (CD34^+^ Flt3^+^) may explain the elevated production of peripheral blood lymphocytes in *Suz12^Plt8/+^ c-Mpl^+/+^* mice.

The functional activity of long-term repopulating stem cells was measured in *Suz12^Plt8/+^* mice by competitive and serial transplantation of bone marrow into lethally irradiated recipients (see Materials and Methods). Both platelet and white blood cell counts were modestly elevated in recipients of *Suz12^Plt8/+^ c-Mpl^+/+^* marrow relative to controls, which recapitulated results seen in unmanipulated *Suz12^Plt8/+^ c-Mpl^+/+^* mice (Table S2) and demonstrated that the phenotype was intrinsic to the bone marrow. Platelet count was similar in recipients of *Suz12^Plt8/+^ c-Mpl^–/–^* and *Suz12^+/+^ c-Mpl^–/–^* marrow, which is likely due to the low representation of *c-Mpl^–/–^* cells in these animals (Table S2).

**Figure 6:**
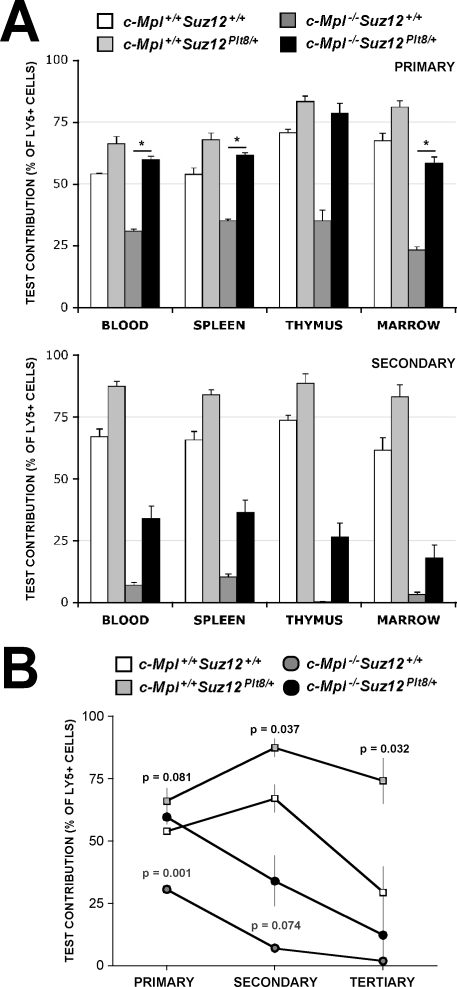
Suz12 Deficiency Enhances HSC Activity in Competitive Transplantation Assays (A) Irradiated recipients (*CD45^Ly5.1^*) were transplanted with an equal number of bone marrow cells from a test animal (*CD45^Ly5.2^,* either *Suz12^Plt8/+^* or *Suz12^+/+^*) and a wild-type competitor (*CD45^Ly5.1^*, *Suz12^+/+^*, and equivalent *Mpl* genotype). Peripheral blood and other tissues were collected and stained with antibodies to CD45^Ly5.1^, CD45^Ly5.2^, and various lineage markers to measure the contribution of the test marrow to hematopoiesis. *Suz12^Plt8/+^* cells made a greater contribution than competitor cells (*Suz12^+/+^*) on both a *c-Mpl^+/+^* and a *c-Mpl^–/–^* background. Serial transplantation was performed at 12–20 wk. Primary (top panel) and secondary recipients (bottom panel) were analysed 3 mo after transplantation. Each column is the average of 3–4 test marrows transplanted into 5 recipients. An asterisk denotes statistical significance (*p* < 0.004) corrected for multiple testing. (B) The representation of Ly5.2^+^ test cells in the peripheral blood is plotted in primary, secondary, and tertiary recipients. Comparisons were made between mice with matched *c-Mpl* genotypes (*p*-values are shown in gray for *c-Mpl^–/–^* and in black for *c-Mpl^+/+^*).

*Suz12^Plt8/+^* bone marrow made a greater contribution to hematopoietic tissues than the wild-type competitor cells, irrespective of the *c-Mpl* genotype (Figure 6A). This difference was most apparent on the *c-Mpl^–/–^* background, where the increase was statistically significant in the peripheral blood, spleen, and bone marrow. The low contribution of *c-Mpl^–/–^* bone marrow to hematopoiesis, even in the presence of a *c-Mpl^–/–^* competitor, is most likely due to compromised competition with residual host-derived wild-type marrow. This effect is compounded in recipients of secondary transplants, with *Suz12^Plt8/+^ c-Mpl^–/–^* cells contributing 18% of the bone marrow, whereas *Suz12^+/+^ c-Mpl^–/–^* cells represented just 3%. Although their contribution to hematopoiesis was elevated, *Suz12^Plt8/+^* HSCs were not rapidly exhausted and continued to contribute effectively to hematopoiesis in tertiary recipients (Figure 6B). Previous reports have highlighted a critical role for Ezh2 and the PRC2 complex during B cell maturation [27]; however, the representation of *Suz12^Plt8/+^* cells was consistent across various cell lineages, which included B cells, T cells, granulocytes, and macrophages. This suggested that the *Plt8* mutation does not impair differentiation (unpublished data).

### *Suz12* Knockdown in Primary Bone Marrow Exacerbates the *Plt8* Phenotype

The phenotype of *Suz12^Plt8/+^* mice reflects the effect of partial (heterozygous) loss of Suz12 function. To gain further insight into the role of Suz12 in hematopoiesis, we used short hairpin RNA (shRNA)-mediated silencing to more profoundly impair *Suz12* expression.

Retroviral shRNA constructs were designed to target two core components of PRC2 (*Suz12* and *Ezh2*) or a nonspecific sequence (Nons), and they were validated in the GATA1^–^ megakaryocyte-erythroid (G1ME) cell line [51]. A 70% reduction in *Suz12* mRNA was observed in cells that expressed shRNA-Suz12, and a similar reduction in expression was obtained with the construct that targeted *Ezh2* (Figure S2A). Analysis of protein expression revealed a dramatic reduction in Suz12 protein levels in cells that expressed shRNA-Suz12 (Figure S2B). Ezh2 expression and H3K27-3Me levels were also reduced in these cells, which confirmed that PRC2 function was greatly impaired. H3K27-3Me levels were similarly reduced in cells that expressed shRNA-Ezh2 but not in cells that express shRNA-Nons.

We next determined the effect of shRNA-mediated depletion of *Suz12* in HSCs. To perform this experiment, *CD45^Ly5.1^* recipient mice were transplanted with *CD45^Ly5.2^* bone marrow that had been infected with either the MSCV LTR-miR30-SV40 GFP (LMS)-Nons or the LMS-Suz12 retrovirus. The proportion of virally transduced cells (Ly5.2^+^ GFP^+^) was determined prior to transplant and then monitored in primary and secondary recipients.

**Figure 7:**
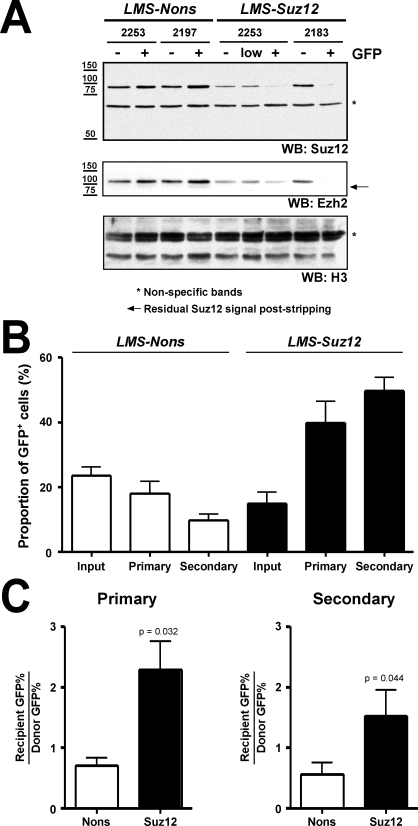
Inhibition of Suz12 by shRNA-Mediated Silencing Elevates HSC Contribution to Hematopoiesis Bone marrow extracted from 5-FU–treated mice was infected with either the LMS-Nons or the LMS-Suz12 virus and transplanted into recipient mice. Three independent infections were performed, and in each case, infected cells were transplanted into five recipient animals. A selection of primary recipients (9–11) were used as donors for secondary transplants, in each case these cells were transplanted into 3–5 recipient mice. (A) Thymocytes were isolated from primary recipients 12 wk after transplantation and fractionated based upon expression of GFP (+ or –); low or intermediate populations were detected in some mice (low). Protein lysates were prepared from sorted cells and Western blotting was performed to detect expression of Suz12, Ezh2, or histone H3. Nonspecific bands have been marked (*) and an arrow is used to denote residual Suz12 signal that persisted after the membrane was stripped and reprobed. (B) The frequency of cells that carried the virus (GFP^+^) was monitored prior to transplantation (Input) and at 8–12 wk after transplantation in primary or secondary recipients. (C) The representation of GFP^+^ cells was compared between donor and recipient populations and a ratio calculated (recipient GFP%/donor GFP%). Equal representation in recipient and donor populations would result in a ratio of 1.0. The representation of cells infected with LMS-Suz12 continued to increase over the course of the experiment, whereas the representation of LMS-Nons cells remained constant. Data show the mean and standard error. Statistical significance was assessed using an unpaired *t*-test.

Thymocytes and splenocytes isolated from primary recipients were used to verify the reduction in Suz12 expression in vivo. Within the thymus, Suz12 protein expression was specifically reduced in cells infected with the LMS-Suz12 virus (Ly5.2^+^ GFP^+^) (Figure 7A). Ezh2 protein levels were also reduced in these cells (Figure 7A), which is consistent with results obtained in G1ME cells. The expression of Suz12 and Ezh2 was not altered in Ly5.2^+^ GFP^+^ cells isolated from recipients of marrow infected with the LMS-Nons construct. Similar results were obtained when the level of *Suz12* mRNA was quantified in these cells (Figure S3).

The contribution of cells infected with LMS-Suz12 to recipient hematopoiesis increased steadily over the course of the experiment; they represented 15.2% of the donor population at the time of transplantation, which increased to 39.8% in primary recipients and to 49.7% in secondary recipients (Figure 7B). This increase was specifically associated with Suz12 deficiency, as cells transduced with the LMS-Nons vector were present at a gradually reducing frequency at infection, in primary recipients, and in secondary recipients (23.7%, 18.1%, and 9.8%, respectively), which is consistent with results using unmanipulated wild-type bone marrow (Figure 7B). In an attempt to standardise the three experiments and account for differences in the absolute number of GFP^+^ cells in each donor, a ratio was calculated using paired donor and recipient data. These data demonstrate that the representation of cells infected with the LMS-Suz12 construct increased by approximately 2-fold upon transplantation into primary recipients, and a similar increase was observed upon transplantation into secondary recipients (Figure 7C), whereas the representation of LMS-Nons–infected cells remained relatively constant. Changes within the progenitor compartment were also greater than those observed in *Suz12^Plt8/+^* mice; as both blast colony formation and megakaryocyte progenitor number was elevated in recipients of marrow infected with the LMS-Suz12 construct (Table 3).

**Table 3:**
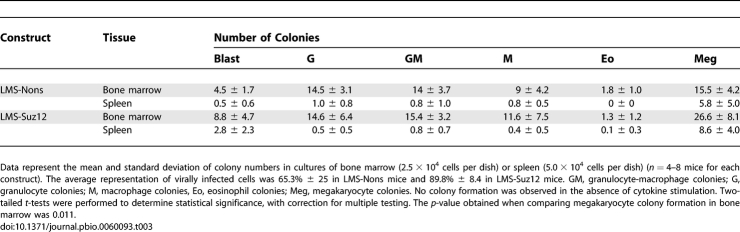
Hematopoietic Progenitors in *Suz12* Knock-down Bone Marrow

### Identification of Genes Sensitive to PRC2 Dysfunction in HSCs

We next analysed gene expression changes in hematopoietic progenitors isolated from *Suz12^Plt8/+^* mice and recipients of LMS-Suz12–infected bone marrow. Global gene expression was examined in LSK cells from the bone marrow of *Suz12^Plt8/+^ c-Mpl^+/+^* and *Suz12^+/+^ c-Mpl^+/+^* mice. Expression differences between the two genotypes were modest, which was consistent with studies performed with *Suz12*-deficient embryonic stem (ES) cells [9,52]. We selected 100 genes with the most significant differences for further analysis (LSK top 100, Table S3). In addition, we isolated Ly5.1^–^ Lin^–^ c-Kit^+^ (LK) cells from mice reconstituted with LMS-Suz12– or LMS-Nons–infected bone marrow. Sca-1 expression was negligible in the Lin^–^ fraction of the bone marrow of secondary transplant recipients, despite the long-term repopulating capacity of these cells; therefore, the progenitor-enriched LK cell population was used for gene expression analysis. We selected the 100 genes that changed most significantly, and were not viral-encoded, for further analysis (LK top 100, Table S4 and Text S1).

**Figure 8:**
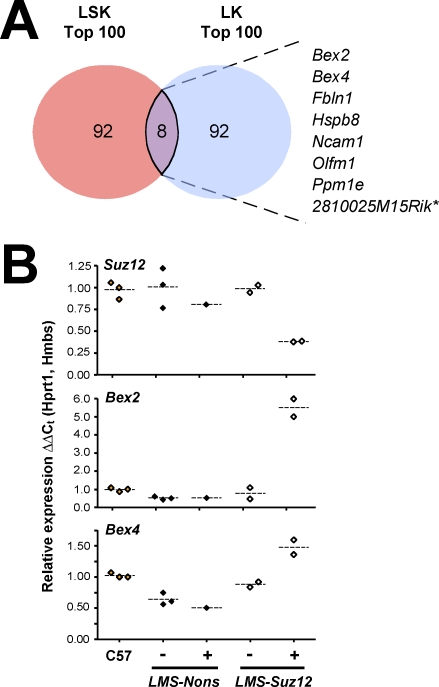
An Analysis of Gene Expression Changes in PRC2 Deficient Hematopoietic Progenitors Cells (A) A direct comparison identified eight genes that were significantly up-regulated in both *Suz12^Plt8/+^* LSK cells (LSK top 100) and in LK cells that expressed the *Suz12* shRNA (LK top 100). This included *Brain expressed 2* (*Bex2*), *Bex4*, *Fibulin 1* (*Fbln1*), *Heat shock protein 8* (*Hspb8*), *Neural cell adhesion molecule 1* (*Ncam1*), *Olfactomedin 1* (*Olfm1*), and *Protein phosphatase 1E* (*Ppm1e*). (B) Expression changes were confirmed in sorted LK samples using Q-PCR. Samples were sorted based upon expression of GFP (+/–), and expression values were compared to LK cells isolated from wildtype C57BL/6 mice (C57).

We analysed the overlap between the LSK and LK top 100 datasets, and we found eight genes that were over-expressed in both *Suz12^Plt8/+^* LSK cells and LK cells deficient in Suz12 compared with controls (Figure 8A), far more than expected by chance (*p* < 0.00001). Real-time quantitative PCR (Q-PCR) was used to confirm results obtained in the microarray and to better quantify the magnitude of the changes in gene expression. Similar to results obtained with thymocytes and splenocytes, *Suz12* expression was markedly reduced in LK cells that express shRNA-Suz12, whereas the expression of *Bex2* and *Bex4* was elevated. It remains to be determined whether the genes deregulated in *Suz12^Plt8/+^* HSCs are direct targets of PRC2.

## Discussion

Using a forward genetics approach, we identified a loss-of-function allele of *Suz12* that suppresses the thrombocytopenia evident in *c-Mpl^–/–^* mice. As well as having an increased platelet count, *Suz12^Plt8/+^ c-Mpl^–/–^* mice display alterations in the number and function of multipotent hematopoietic progenitors and stem cells. Aspects of the *Suz12^Plt8/+^* phenotype were only apparent in the absence of thrombopoietin signalling, which confirmed that *c-Mpl^–/–^* mice provide a sensitised background to detect changes in the progenitor compartment and in the platelet lineage [48]; however, the repopulating activity of HSCs was elevated, irrespective of the *c-Mpl* genotype. The stem cell phenotype was exacerbated when *Suz12* was inhibited by shRNA-mediated silencing, providing independent confirmation that the mutant phenotype is a direct result of impaired Suz12 expression. The identification of the *Plt8* mutation has shown that Suz12 is sensitive to gene dosage within the HSC compartment, an observation that was not appreciated in an earlier loss-of-function study [23], and this lead us to investigate the function of Suz12 and PRC2 during hematopoiesis.

Whether the reduction in Suz12 protein evident in *Suz12^Plt8/+^* mice would affect the stability of the PRC2 complex was unclear. Previous studies have demonstrated that Ezh2, Suz12, and Eed are interdependent, such that a reduction in any one of the PRC2 components negatively affects the stability of the others. This is evident in mice harbouring loss-of-function alleles of PRC2 components [23,53] and in cell lines in which one of the components has been impaired by RNA-mediated silencing [11]. Mice that are heterozygous for the *Plt8* mutation also display decreased levels of Ezh2, suggesting that the reduced expression of Suz12 limits the formation of the PRC2 complex. Similarly, gene dosage effects are evident in mice that carry hypomorphic alleles of *Eed* (*Eed^3354^* or *Eed^1989^*) [21,33,54,55] or a loss-of-function allele of Ezh2 (this study). The discovery that *Ezh2*, *Eed*, and *Suz12* are all haploinsufficient demonstrates that the activity of the PRC2 complex is exquisitely sensitive to alterations in the expression of its components. This is further supported by the observation that the composition and activity of the complex becomes altered when the components are expressed at inappropriate levels [56].

One of our key findings was that *Suz12^Plt8/+^* mice have enhanced HSC activity, which likely accounts for changes that are evident in the peripheral blood. A similar phenotype has been described in mice that carry a hypomorphic allele of *Eed*. *Eed^3354/+^* mice show elevated numbers of multi-potent progenitors in long-term bone marrow cultures [33], and this finding was used to suggest an important function for PRC2 in restricting the proliferation of myeloid and lymphoid progenitors. Our study extends this result and has identified a role for PRC2 in regulating the functional activity of HSCs. The proliferative defects evident in Eed*^3354/+^* mice worsen with age and ultimately progress to leukaemia [33]. Although HSCs derived from *Suz12^Plt8/+^* mice were clearly more competitive than wild-type cells, leukaemia was not observed in *Suz12^Plt8/+^* mice or in recipients of *Suz12^Plt8/+^* bone marrow. It is tempting to speculate that the phenotypic similarities between *Eed^3354/+^* and *Suz12^Plt8/+^* mice result from their common contribution to the PRC2 complex, and that progression to leukaemia in *Eed^3354/+^* mice is a consequence of a greater impairment to PRC2 activity. Elevated platelet counts evident in *Ezh2^+/-^ c-Mpl^–/–^* animals strongly suggest that PRC2 is central to the phenotypic changes in *Suz12^Plt8/+^* mice; however, it remains possible that Suz12 has a functional role that is independent of the complex.

A series of elegant studies have demonstrated that PRC2 functions to maintain the undifferentiated state in embryonic stem cells, and it is quite possible that the complex fulfils a similar role in HSCs. An increased rate of differentiation, in the context of PRC2 deficiency, could explain the elevated contribution of *Suz12^Plt8/+^* HSCs to hematopoiesis and is consistent with the progressive nature of the hematopoietic defects evident in *Eed^3354/+^* mice [33]. Kamminga and colleagues recently demonstrated that exogenous expression of Ezh2 preserves stem cell function during serial bone marrow transplantation and suggested that PRC2 prevents replicative senescence within the HSC compartment [36]. With this in mind, we performed experiments to assess the integrity of homeostatic mechanisms that regulate the HSC pool in *Suz12^Plt8/+^* mice. Although alterations in the number and function of HSCs were detected, several observations suggest that these cells retain their ability to self-renew and do not become senescent prematurely: first, the hematopoietic system is stable in *Suz12^Plt8/+^* mice as they age; second, *Suz12^Plt8/+^* HSCs continue to contribute effectively to hematopoiesis, even after three rounds of transplantation; and third, mice that carry the *Plt8* mutation respond normally to treatment with the cytotoxic agent 5-fluorouracil, which selectively targets cycling cells (unpublished data). It is likely that additional resources, such as conditional targeted alleles, will be required to determine the precise role of PRC2 within the HSC compartment.

To better understand the mechanism by which the *Plt8* mutation influences hematopoiesis, we investigated changes in gene expression associated with PRC2 deficiency. Before this study, knowledge of PRC2 target genes within the hematopoietic compartment was limited. We used shRNA-mediated silencing to impair *Suz12* expression in the erythro-megakaryocytic cell line G1ME and in primary hematopoietic progenitors and stem cells. Global analysis of gene expression by microarray identified several hundred transcripts that were differentially expressed in cells that lacked *Suz12*. The vast majority of these genes showed elevated expression, which supports the prevailing view that PRC2 and H3K27-3Me are required for the maintenance of transcriptional repression (reviewed in [6]). Previous studies have identified genes that are regulated by PRC2 in a variety of different cell types, including mouse and human ES cells, fibroblasts, and numerous cell lines derived from tumours. Our results suggest that PRC2 regulates a distinct set of genes in hematopoietic cells, because very few of the genes identified as mis-regulated in *Suz12*-deficient hematopoietic cells have previously been reported as PRC2 targets [9–11,52].

We identified eight genes that were similarly altered in *Suz12^Plt8/+^* LSK cells and *Suz12*-deficient progenitors (LK cells), demonstrating that some target genes are conserved between stem cells and progenitors. This included the uncharacterised transcript *2810025M15Rik*, which was also up-regulated in *Suz12*-deficient G1ME cells (unpublished data). Other genes were specifically altered within primary progenitor cells, which included a series of genes that are inappropriately expressed in cancer cells. *Bex2*, *Bex4*, and *Fibulin* have been implicated in the progression of various types of cancer—including breast cancer, glioma, and prostate cancer [57–60]—and work with cell lines and primary tumour samples has provided evidence that epigenetic mechanisms contribute to the regulation of these genes. For example, both *Bex2* and *Bex4* become activated when cancer cells are treated with agents that inhibit DNA methylation [58]. Our results suggest that Polycomb group proteins contribute to silencing these genes. A recent study identified a subset of breast cancers that express high levels of *Bex2*, and it will be important to determine whether these tumours display impaired PRC2 function [61]. Tumours that express *Bex2* are highly sensitive to treatment with tamoxifen, and the inhibition of PRC2 may represent a mechanism to promote *Bex2* expression. Few studies have addressed the role of these genes in leukaemogenesis, yet it has been shown that *Bex2* is highly expressed in acute myeloid leukaemia samples that carry activating translocations in the trithorax group gene, *Mixed lineage leukaemia* (*Mll*) [62,63]. *Bex2* expression appears to be highly responsive to changes that disrupt the balance between Polycomb and trithorax complexes. PRC2 has also been implicated in the development of acute promyelocytic leukaemia via direct interaction with the oncogenic fusion protein PML-RAR [64]. Our results suggest that PRC2 may contribute to leukaemogenesis by directly silencing tumour suppressor genes.

Modulation of PRC2 complex, either through inhibition or enhancement of complex activity, has distinct consequences for the behaviour of HSCs. Major impairment of the complex is associated with defective maturation in lymphoid cells and leukaemia, whereas the modest reduction in complex activity in *Suz12^Plt8/+^* mice enhances blood cell production and the performance of HSCs during transplantation. Our data support an important role for PRC2 in regulating gene expression during hematopoiesis. A more detailed knowledge of PRC2 target genes within the HSC compartment, and their response to altered expression of PRC2 components, will enable a better understanding of the role of the complex during development and in disease.

## Materials and Methods

### ENU mutagenesis screen and genetic mapping.

*c-Mpl^–/–^* mice used in this study were maintained on an inbred C57BL/6 background [41]. Male *c-Mpl^–/–^* mice were injected with 200–400 mg/kg N-ethyl-N-nitrosourea (Sigma), which was dissolved in ethanol and diluted in sodium citrate buffer (100 mM sodium dihydrogen phosphate, 50 mM sodium citrate). ENU-treated mice were mated to *c-Mpl^–/–^* females to produce first-generation (G_1_) mice for analysis. At 7 wk of age, G_1_ mice were bled, and platelet counts were measured using an Advia 120 automated haematological analyser (Bayer). G_1_ mice with elevated platelet counts (>300 × 10^6^/ml) were bred with *c-Mpl^–/–^* mice to test for heritability. Using this approach, the PLT8 pedigree was established, in which approximately 50% of mice showed elevated platelet count consistent with a dominant mode of inheritance.

Progeny-tested mice, with inferred genotype C57BL/6 *Mpl^–/–^ Plt8/+*, were crossed with 129/Sv *Mpl^–/–^ +/+* mice to produce an F_1_ population. F_1_ mice with high platelet counts (>150 × 10^6^/ml) were intercrossed to generate F_2_ mice for mapping. DNA was isolated from 90 F_2_ mice and a genome-wide scan was performed with polymorphic microsatellite markers. A candidate interval for *Plt8* was identified between *D11MIT245* and *D11MIT120* on Chromosome 11 (from base pair 77,045,359 to 83,660,314). Additional SSLP markers were designed using PRIMER3 software available through the Whitehead Institute for Biomedical Research (http://frodo.wi.mit.edu/). A further 531 F_2_ mice were genotyped, and the candidate interval was refined to 1.4 Mbp between *CAR28* and *CAR48* (from base pair 79,314,902 to 80,704,402). Mapping and sequencing primers are included as supplementary information.

### Genomic PCR and DNA sequencing.

Genomic DNA was isolated from affected PLT8 mice and exons were amplified by PCR. PCR was carried out with Platinum Taq polymerase (Invitrogen) in buffer supplied by the manufacturer. Reactions contained approximately 20 ng of template DNA, 2.5 mM MgSO_4_, 50 μM dNTPs, 2 units of polymerase, and 10 pmol of each primer. PCR products were treated with ExoSAP-IT (USB Corporation) according to the manufacturers instruction, and sequenced using the Big Dye Terminator V3.1 sequencing kit (Applied Biosystems). Sequencing reactions were centrifuged through G-50 sephadex columns (GE Healthcare) to remove additional dye products, before processing on an ABI 3700 sequence analyser (Applied Biosystems).

### Haematological analysis.

Manual or automated counts were performed on blood collected from the retro-orbital plexus into sample tubes coated with EDTA (Sarstedt, Germany). In vitro colony assays were used to characterise hematopoietic progenitors as described [41]. Bone marrow (2.5 × 10^4^ cells) or spleen cells (5 × 10^4^ cells) were cultured in 1 ml of 0.3% agar in DMEM supplemented with 20% (v/v) FCS and various recombinant cytokines as defined in the text.

CFU-S were enumerated 12 d after transplantation of donor bone marrow (1.5 × 10^5^ *c-Mpl^–/–^* cells or 7.5 × 10^4^ *c-Mpl^+/+^* cells) into lethally irradiated recipients. Spleens were fixed in Carnoy's solution (60% (v/v) ethanol, 30% (v/v) chloroform and 10% (v/v) glacial acetic acid).

Competitive transplantation studies were performed using *CD45^Ly5.2^* donor animals and *CD45^Ly5.1^* recipients. In each experiment, 1 × 10^6^ test cells (*CD45^Ly5.2^*) were transplanted into lethally irradiated *CD45^Ly5.1^* recipients (5 per donor marrow), with an equal number of *CD45^Ly5.1^* competitor cells. Competitor cells were matched by *c-Mpl* genotype, because *c-Mpl^–/–^* HSCs are rapidly out-competed by *c-Mpl^+/+^* cells [45]. Peripheral blood was analysed at 28 d and at 56 d post transplant, and after 3 mo, bone marrow, spleen, thymus, and peripheral blood were analysed and serial transplantations were performed. In each case, the representation of test and competitor (*CD45^Ly5.2^* and *CD45^Ly5.1^*) was measured in B cells (B220^+^), T cells (CD4^+^, CD8^+^), and in macrophages and neutrophils (Gr1^+^/Mac1^+^). Bone marrow from primary recipients was pooled within each donor group for use in secondary transplants, and the representation of *CD45^Ly5.2^* cells was measured before transplantation. For secondary transplants, 2 × 10^6^ test cells were injected into each *CD45^Ly5.1^* recipient, and tertiary transplants were performed in the same manner.

### Analysis of DNA content and nuclear ploidy.

Bone marrow was isolated into CATCH buffer (Phenol-red free, Ca^2+^-free Hank's balanced salt solution with 3% (w/v) BSA, 1.3 mM sodium citrate, 1 mM adenosine, 2 mM theophylline and 3% (v/v) FCS), and stained with FITC-conjugated anti-CD41 antibody (BD Biosciences). Samples were then treated with concentrated propidium iodide (0.05 mg/ml in 3.4 mM sodium citrate) for 1 h. Cells were washed in CATCH buffer, and aggregates were removed by passage through a 100-μm sieve. Samples were then treated with 50 μg/ml RNAse H (Promega) at room temperature, before analysis on a FACScan 2 flow cytometer (BD Biosciences).

### Western blotting and antibodies.

Protein lysates were prepared from primary tissues, or cell lines, in RIPA buffer (1% (v/v) Nonidet P-40, 0.1% (w/v) SDS, 0.5% (w/v) sodium deoxycholate, 150 mM NaCl, 50 mM Tris.HCl pH 7.5) supplemented with protease inhibitors (Roche Diagnostics). 293T cells grown in DMEM with 10% (v/v) FCS were transfected with expression constructs using FuGENE-6 reagent (Roche Diagnostics). Cells were lysed after 48 h and proteins were separated by SDS-PAGE. Protein was transferred to a PVDF membrane and blotted with antibodies to detect Suz12, Ezh2, Histone 3, H3K27-3Me (Upstate), Akt (Cell Signaling) or the FLAG-epitope (M2) (Sigma).

### Retrovirus production and shRNA-mediated knock-down.

Retroviral supernatants were prepared by transient transfection of 293T cells with plasmids that encode viral envelope proteins and a specific LMS/LMP knock-down vector. shRNAmir constructs (pSM2) that target *Suz12* (A: V2MM_96046 or B: V2MM_196969), *Ezh2* (A: V2MM_35988 or B: V2MM_25325), and a nonspecific sequence (Nons) were obtained from Open Biosystems, and the hairpin sequence was subcloned into the LMS/LMP vectors [65,66]. The LMS/LMP vectors drive expression of a modified micro RNA (mir30 backbone) with selectable markers EGFP or EGFP/puromycin, respectively.

293T cells were transfected using the calcium phosphate precipitation method. 293T cells were treated with 25 μM chloroquine for 30 min prior to transfection in DMEM with 10% (v/v) FCS. The precipitated DNA was added dropwise to the cells, and the media was changed after an 8-h incubation. Media was replaced with Iscove's modified Dulbecco's medium (IMDM) with 10% (v/v) FCS after 24 h, and viral supernatants were harvested the following day.

### Bone marrow infections.

C57BL/6 (*CD45^Ly5.2^*) mice were treated with a single dose of 150 mg/kg 5-fluorouracil (5-FU) (ONCO-TAIN, Mayne Pharmaceuticals) by intra peritoneal injection. After 5 d, bone marrow was collected from femurs and tibias into PBS with 10% (v/v) FCS. Red blood cells and dead cells were removed by centrifugation through Ficoll-Paque (GE Healthcare). Cells were washed once with PBS, and resuspended in IMDM supplemented with 10% (v/v) FCS and cytokines (10 ng/ml IL-6, 5 ng/ml IL-3, 50 ng/ml Flt3 ligand, and 50 ng/ml SCF). Cells were grown overnight at 37 °C in a humidified atmosphere with 10% CO_2_ in air. Retroviral supernatants were applied to culture dishes pre-treated with RetroNectin (Takara Biosciences), and centrifuged at 4000*g* for 1 h at 4 °C. Bone marrow cells were co-cultured with the virus in the presence of polybrene (4 μg/ml) for 24 h to allow for infection. Cells were washed out of polybrene-containing medium into fresh medium, and incubated for 24 h. Cells were removed from dish and washed twice in BSS 3% FCS before being counted. These cells were used to reconstitute lethally irradiated *CD45^Ly5.1^* recipients; approximately 5–10 × 10^5^ viable cells were injected into each recipient.

### RNA extraction and Q-PCR.

Total RNA was extracted from tissues or cell lines using Trizol reagent (Invitrogen), and reverse transcribed with an oligo-dT primer using Superscript-III Reverse Transcriptase according to the manufacturers instructions (Invitrogen). For sorted cell populations, RNA was prepared using RNeasy mini purification columns (Qiagen). Q-PCR reactions were set up to quantify expression of mouse *Suz12*, *Ezh2*, *Eed*, *Hprt1, Bex2, Bex4, and Hmbs* using specific pre-designed Taqman gene expression assays (Mm01304152_m1, Mm00468449_m1, Mm00469651_m1, Mm00446968_m1, Mm02528127_s1, Mm02376173_g1, and Mm00660262_g1, respectively) (Applied Biosystems). Typically, PCR reactions were performed in 10 μl volume, and included the following: 1 μl of cDNA, 0.5 μl pre-designed assay mix (primers and sequence specific probe), 3.5 μl H_2_O, and 5 μl of 2x Taqman Universal Master Mix (Applied Biosystems). All Q-PCR reactions were performed on the ABI 7900 HT real-time PCR platform (Applied Biosystems). Ct values were derived using SDS2.2 software (Applied Biosystems), and relative gene expression was calculated using the 2^−ΔΔCt^ method [67].

### Antibodies for flow cytometry.

Fluorophore- or biotin- conjugated antibodies directed against mouse CD4 (clone GK1.5), CD43 (clone 57), CD8 (clone 53–6.7), c-Kit (clone 2B8), Flt3 (CD135) (clone A2F10.1), IgD (clone 11–26c.2a), IgM (clone II/41), CD45^Ly5.1^ (clone A20), CD45.2^Ly5.2^ (clone 104), Sca-1 (clone D7), Ter119 (clone TER-119), Thy1.2 (CD90.2) (clone 53–2.1), and Rat Ig (clone MRK-1) were obtained from Pharmingen. Anti-CD34 (clone RAM34) was obtained from eBioscience, and goat anti-rat IgG was obtained from Southern Biotech. Rat monoclonal antibodies against the mouse antigens CD3 (clone KT3–1.1), CD19 (clone ID3), B220 (clone RA3-6B2), CD11b (clone M1/70), Gr1 (clone IA8), CD2 (clone RM2.1), CD8 (clone 53–6.7), Ter119 (clone TER-119) were prepared in our own laboratory.

### Purification of LSK or LK cells.

Bone marrow was harvested from 7–12-wk-old C57BL/6 mice, or secondary transplant recipients of LMS-infected bone marrow, 4–6 mo post-transplant. Live nucleated cells were purified by centrifugation in Nycodenz medium (Axis-Shield) with a density of 1.086 g/cm^3^. These cells were incubated with a cocktail of monoclonal antibodies against the lineage markers CD3, CD19, B220, CD11b, Gr1, CD2, CD8, and Ter119 prepared in our own laboratories, then mixed with BioMag goat anti-rat IgG beads (Qiagen). Lin^+^ cells were depleted using a Dynal MPC-L magnetic particle concentrator (Invitrogen). Remaining cells were stained with fluorophore-conjugated anti-Rat Ig antibodies to allow residual Lin^+^ cells to be gated out, then with monoclonal antibodies to Sca-1, c-Kit, CD34, Flt3, and CD45^Ly5.1^ (where applicable). Cells were flow sorted on a FACSDiva, FACSAria (BD Biosciences), or MoFlo (Dako).

### LSK and LK global gene expression analysis.

RNA extracted from 50,000–500,000 LSK or LK cells (Lin^–^, Ly5.1^–^, c-Kit^+^) was labeled, amplified, and hybridised to Illumina MouseWG-6 V1.1 Expression BeadChips according to Illumina standard protocols. Samples were processed at the Queensland Institute of Molecular Biology, Brisbane, Australia, and the Australian Genome Research Facility, Melbourne, Australia. Each sample was derived from bone marrow LSK or LK cells from at least six donor mice. A total of 12 LSK arrays were performed (9 *Suz12^+/+^* and 3 *Suz12^Plt8/+^*). Data were analyzed in R and subjected to variance stabilising transformation and quantile normalization. Linear modelling using an empirical Bayes approach, including a batch factor, was applied to the data [68]. Data was corrected for multiple testing using Benjamini and Hochberg correction.

For the combinatorial comparison with LSK and LK datasets, all probesets were considered (irrespective of expression level). Data were normalized and corrected for multiple testing as above. A more detailed description of microarray data treatment is provided (Text S1). Microarray data is available in MIAME-compliant form at Array Express (www.ebi.ac.uk/arrayexpress/) under accession (E-TABM-380).

## Supporting Information

Figure S1 Detection of Suz12 Deletion Mutants A schematic representation of Suz12 truncation mutants is shown (at left). Expression constructs were generated to direct synthesis of Suz12 protein that lacked the N terminus (ΔN), or that lacked both the N and C terminus (ΔNΔC). Additional transfection controls included a construct that directed expression of enhanced green fluorescent protein (EGFP) and a construct encoding Flag-tagged Ezh2. Western blotting demonstrated that the Suz12 polyclonal antibody bound to endogenous full-length Suz12 (marked by asterisks), as well as to the truncated proteins (open circles). Membranes were probed with the Flag antibody to identify epitope tagged exogenous proteins (bottom panel).
(213 KB TIF)

Figure S2 Validation of Suz12 and Ezh2 shRNAs in G1ME Cells RNA and protein levels of PRC2 components were measured in G1ME cells infected with vector alone (LMP) or with viruses that contained an shRNA targeted at a non specific sequence (Nons), *Suz12*, or *Ezh2*.
(A) Gene expression was quantified using Taqman gene expression assays and is shown relative to a reference sample prepared from pooled G1ME cells (Cal). *Hprt1* was used to normalise samples for variation in cDNA concentration. Values represent the mean derived from three independent infections. To determine whether the targeted mRNA was reduced, samples were compared to the Nons control. An analysis of variance (ANOVA) was performed followed by a Bonferroni post-hoc test (*p < 0.01, **p < 0.001).
(B) Protein expression was analysed in stably infected G1ME cell lines. Western blotting was performed to detect expression of Suz12, Ezh2, or H3K27-3Me. H3 was used to verify equal loading.
(171 KB PDF)

Figure S3 Confirmation of Reduced *Suz12* Expression in Primary Cells Thymocytes and splenocytes were isolated from primary recipients 12 wk after transplantation and fractionated based upon expression of GFP (+ or –). cDNA was prepared from sorted cells, and Q-PCR was performed to detect expression of *Suz12*, *Ezh2*, and the house keeping gene *Hprt1*. *Suz12* expression was clearly reduced in cells that expressed the Suz12-shRNA, but not in cells infected with the Nons construct. Although the level of Ezh2 protein was lower in thymocytes deficient in Suz12, this was not reflected in lower levels of Ezh2 transcript.
(34 KB PDF)

Table S1 The *Plt8* Mutation Fails to Complement a Loss-of-Function Allele of *Suz12*
Data represent the number of pups of each genotype derived from mating *Suz12^Plt8/+^* mice with *Suz12^502gt/+^* partners. Data show the number of viable pups present at 3 wk of age. The total number of viable pups was used to calculate expected frequencies. Chi-squared tests were performed (*p < 0.05).
(21 KB DOC)

Table S2 Peripheral Blood Profile of Secondary Transplant Recipients Means ± standard deviations are shown (*n* = 3–4 recipient groups per test bone marrow, each recipient group had 4–5 mice). Two-tailed *t*-tests were performed to determine statistical significance, with correction for multiple testing. Comparisons were made between *Suz12^+/+^* and *Suz12^Plt8/+^* genotypes on *c-Mpl^–/–^* and *c-Mpl^+/+^* backgrounds. No statistically significant differences were observed. The *p*-value obtained when comparing platelet count on *c-Mpl^+/+^* background was 0.04.
(23 KB DOC)

Table S3 Gene Expression Analysis *Suz12^Plt8/+^* LSK Cells Gene expression analysis was performed with wild-type and *Suz12^Plt8/+^* LSK cells. The top 100 genes (sorted by *p*-value) are listed along with their GenBank IDs, fold change, and extended gene name. Analysis was performed with Illumina MouseWG-6 V1.1 Expression BeadChips according to Illumina standard protocols.
(38 KB XLS)

Table S4 Gene Expression Analysis LMS-Suz12 LK Cells Ly5.1^–^ Lin^–^ c-Kit^+^ (LK) GFP^+^ or GFP^–^ cells were sorted from recipients of LMS-infected bone marrow 4–6 mo post-transplant. Gene expression analysis was performed to identify genes specifically altered in cells that expressed the *Suz12* shRNA. The top 100 genes (sorted by *p*-value) are listed along with their GenBank ID, fold change, and extended gene name. Analysis was performed with Illumina MouseWG-6 V1.1 Expression BeadChips according to Illumina standard protocols.
(42 KB XLS)

Text S1 LSK and LK Array Analysis (42 KB DOC)

### Accession Numbers

The Entrez Gene ID (http://www.ncbi.nlm.nih.gov/sites/entrez?db=gene) for genes and gene products discussed in this paper are as follows: *Bex2* (GI:12069), *Bex4* (GI:19716), Bmi-1 (GI:12151), *c-Mpl* (GI:17480), *c-Myb* (GI:17863), *Cyclin-dependent kinase 5 activator 1 precursor* (GI:12569), *Cytokine receptor-like factor 3* (GI:54394), Eed (GI:13626), Ezh2 (GI:14056), *Fibulin* (GI:14114), *Mll* (GI:214162) *Rhomboid veinlet-like protein 4* (GI:246104), *Ring finger protein 135* (GI:71956), *Suz12* (GI:52615), Tpo (GI:21832), and *Zinc finger protein 207* (GI:22680).

## Abbreviations

CFU-S: colony-forming units spleen
Eed: Embryonic Ectoderm Development protein
ENU: N-ethyl-N-nitrosourea
Ezh2: Enhancer of Zeste 2
Flt3: FMS-like tyrosine kinase 3
G1ME: GATA1^–^ megakaryocyte-erythroid
H3K27: histone 3 at lysine 27
H3K27-3Me: tri-methylated H3K27
HSC: hematopoietic stem cell
Lin^–^: lineage marker negative
LK: Ly5.1^–^ Lin^–^ c-Kit^+^
LSK: Lin^–^, Sca-1^+^, c-Kit^+^
PLT8: platelet 8
PRC: Polycomb Repressive Complex
shRNA: short hairpin RNA
*Suz12,*: *Suppressor of Zeste 12;*
Tpo: Thrombopoietin
