# Supplementary material for: Polycomb Repressive Complex 2 (PRC2) Restricts Hematopoietic Stem Cell Activity
Source: PLoS Biol. 2008 Apr 15;6(4):e93. doi: 10.1371/journal.pbio.0060093 (PMC2292752; doi:10.1371/journal.pbio.0060093)
Supplement: Supplementary file 1 [file pbio.0060093.sg002.pdf]

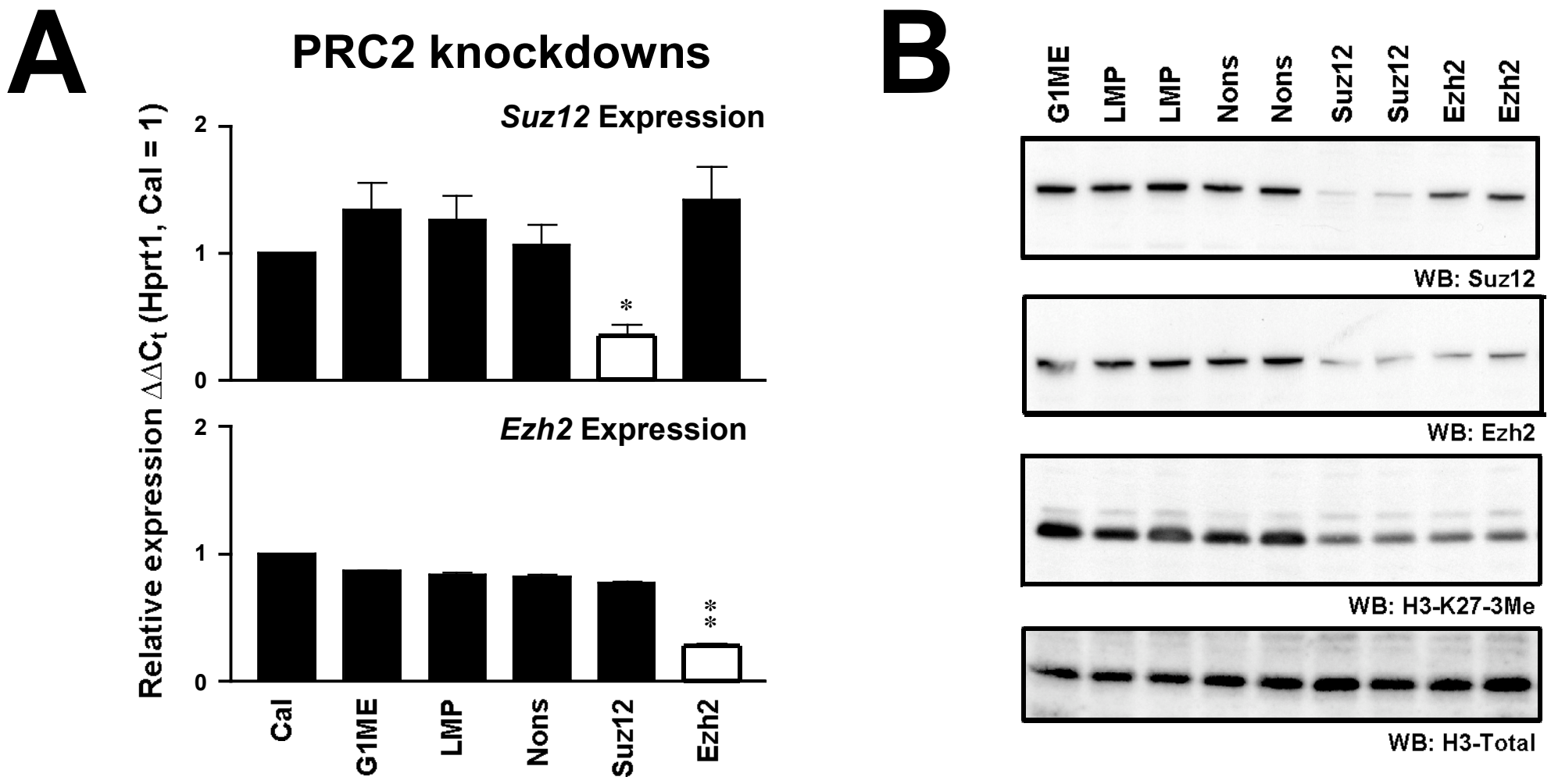

A
PRC2 knockdowns
Suz12 Expression
Ezh2 Expression
Relative expression ΔΔCt (Hprt1, Cal = 1)
2
1
0
Cal
G1ME
LMP
Nons
Suz12
Ezh2
B
G1ME
LMP
LMP
Nons
Nons
Suz12
Suz12
Ezh2
Ezh2
WB: Suz12
WB: Ezh2
WB: H3-K27-3Me
WB: H3-Total
