# Supplementary material for: Polycomb Repressive Complex 2 (PRC2) Restricts Hematopoietic Stem Cell Activity
Source: PLoS Biol. 2008 Apr 15;6(4):e93. doi: 10.1371/journal.pbio.0060093 (PMC2292752; doi:10.1371/journal.pbio.0060093)
Supplement: Supplementary file 2 [file pbio.0060093.sg003.pdf]

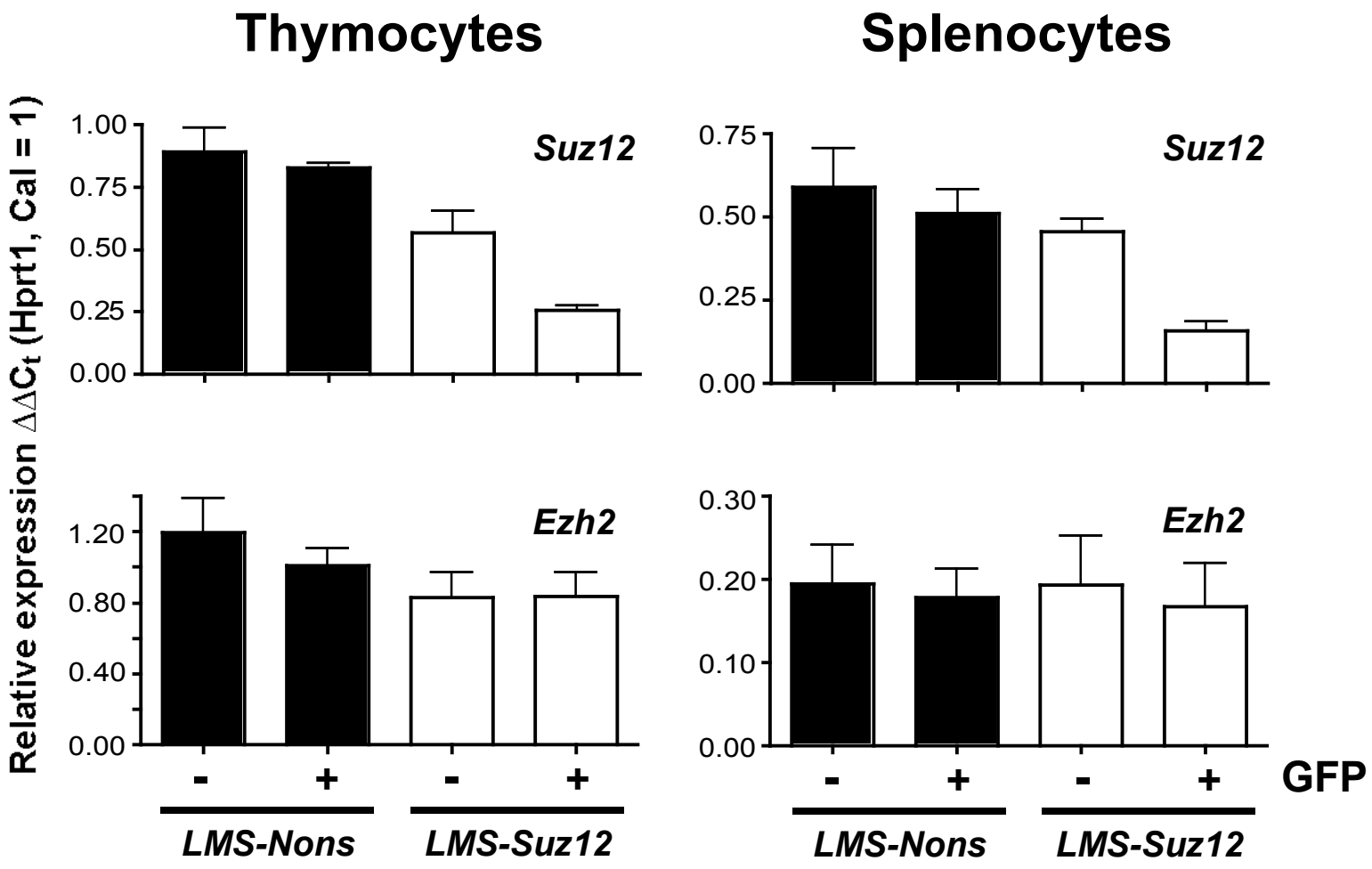
Thymocytes
Splenocytes
Relative expression ΔΔCt (Hprt1, Cal = 1)
Suz12
Ezh2
1.00
0.75
0.50
0.25
0.00
1.20
0.80
0.40
0.30
0.20
0.10
- + - +
GFP
LMS-Nons
LMS-Suz12
