# Supplementary material for: Polycomb Repressive Complex 2 (PRC2) Restricts Hematopoietic Stem Cell Activity
Source: PLoS Biol. 2008 Apr 15;6(4):e93. doi: 10.1371/journal.pbio.0060093 (PMC2292752; doi:10.1371/journal.pbio.0060093)
Supplement: Supplementary file 3 [file pbio.0060093.sd001.doc]

**Supplementary Methods: LSK and LK array analysis**

Ly5.1^-^ Lin^-^ c-Kit^+^ (LK) GFP^+^ or GFP^-^ cells were sorted from recipients of LMS infected bone marrow four to six months post-transplant. Each sample was derived from bone marrow LK cells from at least 6 animals. We prepared 7 samples in total: 3 LMS-Suz12 GFP^+^, 1 LMS-Suz12 GFP^-^, 1 LMS-Nons GFP^+^ and 2 LMS-Nons GFP^-^. Each of these samples was used to prepare RNA, which was labelled, amplified and hybridised to Illumina MouseWG-6 V1.1 Expression BeadChips as described in the Materials and Methods section.

Two comparisons were performed to identify the genes that were differentially expressed between the *Suz12* knockdown and control cells, and to remove all virus specific genes. Firstly, we performed a comparison between LMS-Suz12 GFP^+^ LK cells (n = 3) and a group of either LMS-Suz12 GFP^-^ or LMS-Nons GFP^-^ LK cells (GFP^-^, n = 3), representative of cells that were not infected. The differentially expressed genes were sorted according to p-value. We selected the genes that were more significantly different than genes related to viral infection (e.g. *EGFP*) which we knew to be real differences (unadjusted p-value < 0.007). We performed a second comparison between LMS-Suz12 GFP^+^ LK cells (n = 3) and LMS-Nons GFP^+^ LK cells (n = 1), and used this comparison to validate those genes in the first comparison by selecting only those genes that changed 1.2 fold or more in the same direction. This approach removed all virus-specific genes, and we then selected the 100 validated genes that changed most significantly in the LMS-Suz12 GFP^+^ versus GFP^-^ comparison for further analysis (LK Top 100, Supplementary Table, Supplementary Figure).

We then determined which genes were altered in common between the LSK Top 100 and the LK Top 100 (Fig. 8A), which represents a comparison solely based on statistical significance.

**Supplementary Methods: Genotyping and sequencing primers**

In this section we provide details of primers and PCR conditions used in SSLP genotyping, candidate gene sequencing and mouse strain genotyping:

# SSLP Genotyping

A robotic platform (Packard Instruments, Meridan, USA) was used to set up PCR reactions for genetic mapping. Reactions were performed in an 8 μL volume, which contained ~40 ng template DNA, 0.125 mM dNTPs (Amersham Biosciences, Uppsala, Sweden), 0.8 μl 10x PCR buffer (25 mM MgCl_2_, 100 mM Tris.HCl pH 8.3, 500 mM KCl), 10 pmol of each fluorescent primer (Integrated DNA Technologies, Coralville, USA) and 0.075 μl Taq polymerase. Reactions were performed in 384 well thermocycler using a touchdown PCR protocol. 1 μL of pooled PCR product was mixed with 9.7 μL of HiDi formamide (Applied Biosystems, Foster City, USA) and 0.3 μL PRISM Genescan-500 ROX size standard (Perkin Elmer Life Sciences, Wellesley, USA). Samples were denatured by heating to 95°C for 5 minutes and were then resolved on an ABI 3700 capillary electrophoresis machine. Genescan 3.1 and Genotyper 2.0 software (Perkin Elmer) were used to analyse genotyping data.

The primer sequences for SSLP markers detailed in the text:

| Marker | **Chr.** | **Forward** | **Reverse** |
| --- | --- | --- | --- |
| D11MIT245  D11MIT120  D11MIT322  D11CAR28  D11CAR48  D11CAR9  D11CAR61  D11CAR86 | 11  11  11  11  11  11  11  11 | ATGAGACCATGCTCCTCCAC  CTTCTGATTTCCTCTTGCACG  TGTCCTGCATACCCCTTTG  GGAGCCATTATGGAAACTGC  GGAGATGGTTTGGGATACACA  TGCTAGGAACCAAACCAGGA  TTGAAGGATTTCTGCCCTTA  TGCACAGAGACACAAGCACA | TTGTCCTCTGACCTTCACACC  TGGCATAAGAGACAGGCTCA  GATTAAGTACAGGCAACTGCAGG  GTTTCTTTGAGCATATCCCTGAGAAGGA  GTTTCTTTGAGCATATCCCTGAGAAGGA  GTTTCTTATTCACCACCACACCTGACA  GTTTCTTTATGTGATGCAGGTGCAGTG  GTTTCTTGACTCGCTGGACACACAAAA |

SSLP markers were amplified from genomic DNA using a touchdown PCR protocol:

| Cycle | **Temperature (°C)** | **Time (s)** |
| --- | --- | --- |
| 1 | 94 | 180 |
| 2-6 | 94  65 (-3°C per cycle)  72 | 15  30  30 |
| 7-41 | 94  50  72 | 15  30  30 |

### Candidate gene sequencing

PCR was generally performed in 25 μl reaction volumes, which contained ~50 ng DNA template, 2 mM MgCl_2_, 50 μM dNTPs, 2 Units of Platinum Taq polymerase^TM^ (Invitrogen, Carlsbad, USA) and 10 pmol of each primer in buffer provided by the supplier. Oligonucleotide primers (Geneworks, Adelaide, Australia) were designed to amplify exonic regions for candidate gene sequencing, with each amplicon being 500-600 base pairs in length. Typical PCR conditions were as follows: incubation at 94°C for 3 minutes, followed by 35 cycles of 94°C for 30 seconds, 55°C for 30 seconds and 72°C for 60 seconds. The annealing temperature, extension time and magnesium concentration were altered to optimise amplification of target nucleotide sequences.

Prior to sequencing, PCR products were treated with ExoSAP-IT (USB Corporation, Cleveland, USA) for 15 minutes at 37°C to remove excess dNTPs and oligonucleotide primers. A typical sequencing reactions contained 5 μl of treated PCR product, 4 μl of BigDye Terminator V3.1 mix (Applied Biosystems), 3.2 pmol oligonucleotide primer and buffer provided by the supplier. The reactions were performed in a thermocycler as follows: incubation at 96°C for 2 minutes, followed by 25 cycles of 96°C for 10 seconds, 50°C for 5 seconds and 60°C for 4 minutes. Sequencing reactions were centrifuged through G‑50 sephadex columns (GE Healthcare, Piscataway, USA) at 800 g for 5 minutes in a MultiScreen-HV plate (Millipore, Massachusetts, USA), dried down and reconstituted in 10 μl of HiDi formamide before processing on an ABI 3700 sequence analyser (Applied Biosystems).

Oligonucleotide primers used to amplify and sequence *Suz12*:

| Gene | **Exon** | **Forward** | **Reverse** | **Product (bp)** |
| --- | --- | --- | --- | --- |
| *Suz12* | 1  1  2/3  4  5  6  7  8  9  10  11  12  13  14  15  16 | gtgactgacgggggactct  GGCTCGGAGGAGACACTTTT  gcgggaaaaagattttgtga  tggtgcattgttatggctagt  tctcctctctgaggttttgtttg  ctggagttgaattgggaggt  cacattccattttgtacttgtgg  ttcaagaccagcctgctgta  aagaatgcatttctgggtgtg  ctgcagccccaataatgagt  cccaggattatgtggcgtat  CGCTGACTACAGAGCTGCAA  cgtggtgttgcacaccttt  gagggttcagagagggaggt  acatgcctttggtcccagta  ttgctcggacttgtttgtca | AGGAGGAGGCCGAGTAACTG  tcccaaagcaagtggactct  aaagtgcattttcctgagctg  gttgccacttctccacaggt  tcatcatcgggatatgggta  cttggcttgtctctgcttcc  cagcattcgaagacctggat  tgtgcttcctctcattttgg  aagagggcaccattctcctt  cctcaaacgcagggattaaa  GGTCGTCTCTGGCTTCTGTC  ccactgtaacctgggcagaa  cctcgatttaggctgactgc  ccgaaggtccagagttcaaa  cagtgtcctttggagccact  AACACTGCCTGTTCCCAATC | 508  577  503  552  457  546  495  473  555  463  581  499  576  591  488  557 |

### Mouse strain genotyping

c‑Mpl^-/-^ mice: Three primers were used in a duplex PCR, which allowed for detection of both the wildtype (*c‑Mpl^+^*) and mutant allele (*c‑Mpl^-^*). PCR conditions were as follows: incubation at 94°C for 3 minutes, followed by 35 cycles of 94°C for 30 seconds, 60°C for 30 seconds and 72°C for 60 seconds. The wildtype allele gave a PCR product of 230 bp, whereas the mutant allele gave a product that was 671 bp. PCR products were resolved on a 2% (w/v) agarose gel.

Suz12^502gt^ mice: Three primers were used in a duplex PCR, which allowed for detection of both the wildtype (*Suz12^+^*) and mutant allele (*Suz12^502gt^*). PCR conditions were as follows: incubation at 94°C for 3 minutes, followed by 35 cycles of 94°C for 15 seconds, 60°C for 20 seconds and 72°C for 90 seconds. The wildtype allele gave a PCR product of 230 bp, whereas the mutant allele gave a product that was 671 bp. PCR products were resolved on a 2% (w/v) agarose gel.

Oligonucleotide primers used for PCR genotyping:

| Strain | **Primer** | **Oligonucleotide sequence** | Size (bp) |
| --- | --- | --- | --- |
| *c‑Mpl^-/-^* | Forward  Reverse  Reverse | TCCAAGGTAAAGCACTGAAGTCCA  GTCTCCATGGAGGCTTAGGTGGA  GAAGAGCTTGGCGGCGAATGGGCT | 230  **671** |
| *Suz12^502gt^* | Forward  Reverse  Reverse | GCTGGCCATGGTAGTTTGTT  CCAAACCTGGGTCCTCTGTA  CCTGGCCTCCAGACAAGTAG | 694  **403** |

*Suz12^Plt8^* mice: A custom designed AD assay (Applied Biosystems) was used to genotype the *Suz12^Plt8^* allele. Such assays contain fluorescently labelled probes that hybridise to either the wildtype (VIC) or the mutant allele (FAM) that are coupled to a non-fluorescent quencher. PCR extension from primers flanking the mutation site releases the dye from the quencher, and the level of VIC and FAM fluorescence measured after 40 rounds of PCR. PCR reactions were performed in a volume of 10 μl and contained: ~10 ng genomic DNA, 13 pmol of each primer, 2.5 pmol VIC-probe (for the wildtype allele), 2.5 pmol FAM-probe (for the mutant allele) and 5 μl of 2x Taqman^TM^ Universal Master Mix (Applied Biosystems). All AD reactions were performed on an ABI 7900 HT real-time PCR platform (Applied Biosystems).

Oligonucleotide primers and probes using in allelic discrimination assays:

| Locus/  Mutation | **Primer/**  **Probe** | **Oligonucleotide sequence** |
| --- | --- | --- |
| *Suz12*  *Plt8* | Forward  Reverse  Wildtype  Mutant | CTCTTGTTGTTTTCCCAAAGAATTTGTATC  CAAACAGCATACAGGCATGATTCAT  VIC-TGTCAGCAATAAATCTATTCA  FAM- TCAGCAATAAATTATTCA |

#### Supplementary Methods: Retroviral infection of G1ME cells

Retroviral infections were performed in a similar manner to those with primary cells, with minor modifications. G1ME cells were grown in alpha-MEM (Invitrogen), which was supplemented with 100 μg/ml streptomycin, 100 IU/ml penicillin, 20% (v/v) FCS and 70-100 ng/ml recombinant Tpo (Stachura *et al.* 2006). Approximately 2 x10^6^ viable cells were used in each infection. Cells were transferred to selective media (standard G1ME growth media supplemented with 2 μg/ml puromycin) after 48 h, and were considered stably transduced after two weeks in selective media.
