# Supplementary material for: Polycomb Repressive Complex 2 (PRC2) Restricts Hematopoietic Stem Cell Activity
Source: PLoS Biol. 2008 Apr 15;6(4):e93. doi: 10.1371/journal.pbio.0060093 (PMC2292752; doi:10.1371/journal.pbio.0060093)
Supplement: Supplementary file 4 [file pbio.0060093.st001.doc]

**Supplementary Table 1.** **The *Plt8* mutation fails to complement a loss-of-function allele of *Suz12***

|  |  | **Genotype (*Suz12^502gt/+^* x *Suz12^Plt8/+^*)** | | | |  |
| --- | --- | --- | --- | --- | --- | --- |
| Time point | **Frequency** | ***Suz12^+/+^*** | ***Suz12^502gt/+^*** | ***Suz12^Plt8/+^*** | ***Suz12^Plt8/502gt^*** | **p-value** |
| **Weaning** | **Observed** | 33 | 32 | 35 | **0*** | 2.50 x10^-7^ |
|  | **Expected** | 25 | 25 | 25 | 25 |  |

Data represent the number of pups of each genotype derived from mating *Suz12^Plt8/+^* mice with *Suz12^502gt/+^* partners. Data show the number of viable pups present at three weeks of age. The total number of viable pups was used to calculate expected frequencies. Chi-squared tests were performed (***** represents p < 0.05).
