# Supplementary material for: Polycomb Repressive Complex 2 (PRC2) Restricts Hematopoietic Stem Cell Activity
Source: PLoS Biol. 2008 Apr 15;6(4):e93. doi: 10.1371/journal.pbio.0060093 (PMC2292752; doi:10.1371/journal.pbio.0060093)
Supplement: Supplementary file 5 [file pbio.0060093.st002.doc]

**Supplementary Table 2. Peripheral blood profile of secondary transplant recipients**

|  | **Test Bone Marrow Genotype** | | | |
| --- | --- | --- | --- | --- |
|  | ***c‑Mpl^-/-^*** | | ***c‑Mpl^+/+^*** | |
| Measurement | ***Suz12^+/+^*** | ***Suz12^Plt8/+^*** | ***Suz12^+/+^*** | ***Suz12^Plt8/+^*** |
| Test contribution (%Ly5.2^+^) | 3.16 ± 1.05 | 17.9 ± 5.32 | 61.8 ± 4.97 | 83.3 ± 4.90 |
| Platelet count (x10^-6^/ml) | 813 ± 139 | 802 ± 101 | 778 ± 101 | 1006 ± 97.9 |
| Mean Platelet Volume (fl) | 6.07 ± 0.66 | 5.72 ± 0.55 | 5.41 ± 0.58 | 5.35 ± 0.75 |
| Hematocrit (%) | 45.3 ± 1.56 | 45.7 ± 0.51 | 47.5 ± 0.80 | 46.4 ± 1.20 |
| White cell count (x10^-6^/ml) | 3.58 ± 1.28 | 5.63 ± 2.98 | 9.71 ± 1.35 | 11.6 ± 2.13 |
| Neutrophils (x10^-6^/ml) | 0.70 ± 0.22 | 0.81 ± 0.21 | 0.83 ± 0.08 | 0.75 ± 0.09 |
| Lymphocytes (x10^-6^/ml) | 2.69 ± 1.09 | 4.59 ± 2.71 | 8.60 ± 1.38 | 10.4 ± 2.01 |
| Monocytes (x10^-6^/ml) | 0.09 ± 0.01 | 0.08 ± 0.03 | 0.08 ± 0.01 | 0.11 ± 0.01 |
| Eosinophils (x10^-6^/ml) | 0.07 ± 0.04 | 0.09 ± 0.05 | 0.08 ± 0.01 | 0.13 ± 0.04 |

Means ± standard deviations are shown (n=3-4 recipient groups per test bone marrow, each recipient group had 4-5 mice). Two-tailed t-tests were performed to determine statistical significance, with correction for multiple testing. Comparisons were made between *Suz12^+/+^* and *Suz12^Plt8/+^* genotypes on *c‑Mpl^-/-^* and *c‑Mpl^+/+^* backgrounds. No statistically significant differences were observed. The p-value obtained when comparing platelet count on *c‑Mpl^+/+^* background was 0.04.
